# Chest packing with negative pressure for refractory mediastinal bleeding after cardiac surgery: a low-cost, simple technique as an alternative to vacuum-assisted closure systems in a retrospective study

**DOI:** 10.12701/jyms.2026.43.49

**Published:** 2026-07-27

**Authors:** Hunbo Shim, Seok Soo Lee, Changseok Jeon, Tae Hee Won, Sang-Wan Ryu, JongHyun Baek

**Affiliations:** 1 Division of Cardiac Surgery, University of Rochester Medical Center, Rochester, NY, USA; 2 Department of Thoracic and Cardiovascular Surgery, Changwon Hanmaeum Hospital, Changwon Korea; 3 Department of Thoracic and Cardiovascular Surgery, Incheon Sejong Hospital, Incheon, Korea; 4 Department of Thoracic and Cardiovascular Surgery, Ewha Womans University Seoul Hospital, Ewha Womans University School of Medicine, Seoul, Korea; 5 Department of Thoracic and Cardiovascular Surgery, International St. Mary’s Hospital, Incheon, Korea; 6 Department of Thoracic and Cardiovascular Surgery, Yeungnam University College of Medicine, Daegu, Korea

**Keywords:** Chest packing, Cardiac surgical procedures, Negative pressure wound therapy, Open chest, Postoperative hemorrhage

## Abstract

**Background:**

Refractory mediastinal bleeding after cardiac surgery occasionally requires temporary open sternum and delayed sternal closure. We evaluated the feasibility and short‑term clinical performance of a modified chest‑gauze suction (c‑GSUC) technique that generates negative pressure using readily available materials.

**Methods:**

We retrospectively reviewed 20 patients who underwent chest packing primarily for refractory bleeding following cardiac or thoracic aorta surgery. After routine drainage placement, gauze sponges were packed around the bleeding sites, and an additional temporary chest tube was positioned over the gauze. The open sternotomy was sealed with an adhesive drape and connected to a standard three-chamber drainage bottle to generate negative pressure and collapse the packed gauze.

**Results:**

The median patient age was 57.0 years, and 19 patients (95.0%) underwent aorta-related procedures. Definitive chest closure was achieved within 2 days in 18 patients (90.0%). One patient (5.0%) required emergent washout for cardiac tamponade during open chest management. One patient (5.0%) developed mediastinitis after closure. Two patients (10.0%) died during hospitalization, one from septic shock and the other from intracranial hemorrhage.

**Conclusion:**

Temporary open sternum with negative‑pressure gauze packing using the c‑GSUC technique was feasible in patients with refractory mediastinal bleeding and was associated with acceptable short‑term outcomes. Although less sophisticated than commercial vacuum-assisted closure systems, this simple and inexpensive approach may be a practical alternative in selected cases.

## Introduction

Open chest and delayed sternal closure following cardiac surgery is a well‑established technique used in patients with unstable hemodynamics and/or refractory mediastinal bleeding. This approach allows selective compression of bleeding points [1] while minimizing interference with diastolic filling [2-5]. Despite the correction of coagulation abnormalities and meticulous surgical hemostasis, refractory mediastinal bleeding remains an unavoidable challenge in cardiac surgery. Previous reports indicate that 2% to 4% of adult patients leave the operating room with an open sternum [6-10] and 2% to 6% require re-exploration for postoperative bleeding [11-16]. Although traditional chest packing can effectively control bleeding with relatively low rates of mediastinitis and acceptable survival, many surgeons are hesitant to leave the sternum open because of concerns about infection, thoracic cage instability, and uncertainty regarding the effectiveness of conventional packing techniques.

Negative pressure-generating devices such as vacuum-assisted closure (VAC) systems, initially developed for managing mediastinitis and sternal wound infections, have recently been used to control mediastinal bleeding and hemodynamic instability in patients with open sternum, with reported reductions in infection rates and improved bleeding control [8].

Traditional chest packing has long been used to treat refractory mediastinal bleeding at our institution. This technique was recently modified and standardized to incorporate negative pressure using simple gauze sponges and a chest tube, with the aim of reproducing the mediastinal compression effects of commercial VAC systems at substantially lower costs. In this study, we evaluated the feasibility and practical performance of this modified chest gauze suction (c-GSUC) technique in patients with refractory mediastinal bleeding after cardiac surgery.

## Methods

**Ethics statement:** This study was approved by the Institutional Review Board (IRB) of Ewha University Medical Center (IRB No: 2022 09 035), and the requirement for informed consent was waived.

### 1. Study cohort and inclusion criteria

A total of 1,650 patients underwent cardiac and/or thoracic aorta surgery at our department between January 2010 and August 2022. Among them, 27 (1.6%) left the operating room with an open chest and were initially screened for inclusion.

Three patients required an open chest solely for hemodynamic compromise without significant bleeding and were excluded to focus on the feasibility of the technique for hemostasis. Two patients who underwent descending thoracic aorta surgery via lateral thoracotomy and two patients with missing data were also excluded. Ultimately, 20 patients who underwent chest packing for uncontrolled coagulopathy after cardiac or thoracic aorta surgery via sternotomy were included in the final analysis.

### 2. Decision-making process for implementation of the open chest technique

The surgeon and anesthesiologist jointly determined the presence of refractory bleeding based on clinical presentation and laboratory findings after heparinization reversal [17]. The following criteria were considered: (1) persistent blood pooling in the operative field, despite no identifiable source of surgical bleeding and ongoing correction of coagulopathy. (2) Blood exceeding 600 mL was purified from the cell saver reservoir canister. Notably, at our institution, each 500 mL of hemodiluted collection in the cell saver canister yields approximately 120 mL of purified erythrocytes. This can vary depending on the degree of dilution. (3) Failing to maintain hemoglobin levels corresponding to transfusion quantities by the third serial hemoglobin evaluation (evaluated at 30-minute intervals). For example, based on the average weight of Korean adults (50–70 kg), a transfusion of 250 mL of packed erythrocytes is expected to increase hemoglobin levels by 1.0 g/dL.

### 3. Technical details of the chest gauze suction technique

At our hospital, a novel chest packing technique was protocolized and unanimously used by all surgeons for the management of patients who required the open chest technique during the study period (Fig. 1). After inserting the usual drainage tubes, a minimum number of gauze sponges were packed around the incision and suture sites (anastomosis sites of the arch vessels and aortotomy, left atriotomy, right atriotomy, and cannulation sites) to prevent packed gauze-related tamponade. Without a sternal bridge, two to three additional gauzes were used to cover the entire surgical field. A 32-French regular chest tube was then placed on top of the covered gauze, and the chest tube side hole was covered with additional gauze. The open wound was sealed with an adhesive drape film (Ioban; 3M Healthcare, St. Paul, MN, USA) to ensure airtightness. The temporary chest tube used to collapse the packed gauze was then connected to a separate drainage bottle, distinct from other chest tubes. A traditional three-chamber underwater seal drain system with a wet suction control chamber was used for all chest bottles. This suction, controlled by the level of water in the suction control chamber, was typically set at -20 cmH_2_O to collapse the packed gauze (Fig. 1F).

### 4. Management during open chest

All patients were sedated, immobilized, and mechanically ventilated in the intensive care unit (ICU) until chest closure. Coagulopathy correction was the primary goal of patient management as all patients underwent chest packing for refractory bleeding. Blood products were transfused according to the patients’ coagulation profiles. Prophylactic intravenous vancomycin was administered after the primary surgery. If any infection was detected on culture tests, appropriate antibiotics were administered after consultation with microbiologists. Once the output from all chest tubes subsided, the patients were transferred to the operating room for washout and chest closure. In addition, for patients with hemodynamic instability caused by cardiac tamponade due to ongoing bleeding, bedside clot evacuation was performed as deemed necessary.

### 5. Estimations of initiation and theoretical maintenance costs

Material costs for c‑GSUC were compared with hypothetical costs of applying a VAC system in the same patient group, with emphasis on the expenses required at initiation. As the technique was performed as part of the primary surgery, no additional labor costs were attributed. In most patients, the duration of open chest maintenance was short; therefore, ICU labor costs were excluded.

The c‑GSUC materials included gauze sponges, chest tubes, adhesive drape film, polyvinyl chloride tubing, connectors, and drainage bottles. Hypothetical VAC costs included gauze sponges, a disposable VAC system kit (tubing and covering film), and drainage canisters. The VAC pressure generator was excluded as it is a reusable hospital asset.

Because the duration of c‑GSUC was brief, the direct estimation of daily maintenance costs was not feasible. To provide a practical comparison, we applied daily maintenance costs reported in a prior study using USA hospital data [18], which estimated an average VAC maintenance cost of $111.2 per day and $4.3 per day for gauze‑based suction techniques. These unit costs were multiplied by each patient’s open chest duration to estimate the theoretical total maintenance costs. Given that both cost values were derived deterministically from the same duration variable, these calculations represent illustrative cost estimates rather than independently observed or statistically comparable data.

### 6. Data collection and statistics

Patient demographics, preoperative laboratory values, intraoperative variables, transfusion data, and postoperative outcomes were extracted from electronic medical records. Continuous variables that demonstrated non-normal distribution on the Shapiro-Wilk test were summarized using the median and interquartile range (IQR). Because VAC and c‑GSUC cost estimates were deterministically derived by multiplying each patient’s open chest duration by fixed daily unit costs, no formal statistical comparison was performed for cost analysis. Cost values are presented as illustrative estimates. All statistical analyses were performed using R ver. 4.4.3 (R Foundation for Statistical Computing, Vienna, Austria).

## Results

### 1. Patient demographics and preoperative conditions

The patient characteristics are summarized in Table 1. The median patient age was 57.0 years, and 14 patients (70.0%) were male. Of the 20 patients included in the study, nine had undergone emergency surgeries and 11 underwent elective surgeries.

Six patients (30.0%) were taking aspirin, clopidogrel, or warfarin, and only one patient received dual antiplatelet therapy. According to our institution’s protocol, low-dose aspirin was administered until the day before surgery, and clopidogrel was discontinued at least 5 days before surgery. Warfarin was fully reversed and activated partial thromboplastin time was maintained within 60 to 80 seconds using an intravenous heparin drip in all elective surgeries. Among the nine patients who required emergency surgery, only one (patient #10) received anticoagulation therapy with warfarin. This patient’s international normalized ratio was normalized before the surgery by administering vitamin K and fresh frozen plasma. The anticoagulation laboratory profiles were within the normal range in most patients (Table 2). Nine patients (45.0%) underwent redo surgery at the time of the index surgery.

### 2. Operative details and chest closure

Thoracic aorta-related surgery was the most performed surgical procedure. A total of 19 patients (95.0%) underwent ascending aorta or ascending aorta/arch surgery. An aortic valve procedure was performed in 14 patients (70.0%). Of these patients, seven (35.0%) underwent the Bentall procedure and three (15.0%) underwent valve-sparing root replacement (David procedure). The median surgery, cardiopulmonary bypass, and myocardial ischemia times were 535, 259, and 199 minutes, respectively. Definitive chest closure was performed by postoperative day 2 in most patients (18 patients, 90.0%) with a median of 1.5 days (IQR, 1.0–2.0 days). These procedures were performed mostly in the operating room (Table 3).

The combined procedures in one patient were counted separately for each procedure. Since the aortic valve is manipulated in valve-sparing aortic root replacement and the Bentall procedure, these aortic valve manipulations were counted as aortic valve surgeries. Massive volumes of blood products were transfused during the surgeries, and coagulopathy was aggressively corrected during the open chest in the ICU (Table 4).

### 3. Comparison of estimated procedure costs

The calculated charges for the one‑time application of c‑GSUC amounted to $33.2, whereas the estimated initiation cost for VAC was $79.2. When applying daily maintenance cost values reported in prior literature [18], the theoretical median total cost was $166.8 (IQR, $111.2–$222.4) for VAC and $6.3 ($4.3–$8.5) for c‑GSUC. Because both cost values were derived deterministically by multiplying each patient’s open chest duration by the fixed daily unit costs, these figures represent illustrative cost estimates rather than statistically comparable data (Table 5).

### 4. Hospital course after definitive chest closure

The median durations of ICU and hospital stay were 6 days and 17.5 days, respectively. Wound dehiscence and possible mediastinitis developed in only one patient after delayed chest closure (patient #17). This patient underwent a Bentall procedure for prosthetic valve endocarditis and was administered antibiotics for continuing bacteremia after the surgery. The wound was repaired with simple revision, but subsequent computed tomography revealed a suspicious shadow, possibly an infected fluid collection. Fortunately, the patient was successfully treated with antibiotics.

Two patients had mediastinitis (patients #8 and #18); however, they had pre-existing mediastinitis as a complication of previous interventions when they were transferred from another hospital. Patient #18 experienced wound dehiscence accompanied by purulent discharge 22 days after chest closure. The wound was explored and the VAC system was used for 10 days. The patient was discharged after wound closure. Patient #8 died of septic shock, and together with patient #19, who died of intracranial hemorrhage, were counted as two in-hospital mortalities.

Three patients (15.0%) experienced significant pericardial effusion. Only one of them developed hemodynamically unstable cardiac tamponade with an open chest and required emergent chest washout due to a hematoma compressing the heart (patient #3). However, two patients required separate pericardiostomy for pericardial effusion after chest closure (patients #8 and #15). One patient (patient #2) developed complete atrioventricular block, and a permanent pacemaker was implanted on postoperative day 11 (Table 6).

### 5. Clinical outcomes after discharge

In addition to the two in-hospital deaths, one patient (#3) was sent to a rehabilitation facility because of a cerebrovascular accident for which follow-up information could not be obtained.

Of the 17 patients who were discharged, 12 experienced no events within 1 year after discharge (patients #1, 2, 4–7, 9, 11, 16–18, and 20), including patients #17 and #18, who underwent wound revision prior to discharge. Patient #10 developed right carotid artery stenosis 8 months after discharge, necessitating admission to the neurology step-down unit for medical intervention. This patient did not have any procedure-related complications 1 year postoperatively.

Three patients (#12, #13, and #14) were lost to follow-up after their 3-month follow-up visits, and one patient (#15) was lost to follow-up 8 months postoperatively. No complications were observed in these patients during the observed follow-up period.

## Discussion

Persistent mediastinal bleeding after cardiopulmonary bypass is a life-threatening complication of cardiac surgery. Various factors including technical issues, increased heparin activity, hemodilution, hypothermia, increased fibrinolytic activity, and platelet dysfunction have been associated with increased blood loss after cardiac surgery [19-22]. Despite coagulopathy correction and the use of surgical or chemical hemostasis, continuous bleeding may occur in some cases. In these patients, a temporarily open sternum and cardiac structural compression can be lifesaving. Institutions use different protocols for maintaining an open sternum, including gauze packing, temporary skin closure without sternal approximation, and silicone membrane coverage [2,23-25]. However, concerns remain regarding traditional techniques, including infection risk, thoracic cage instability requiring more invasive respiratory support, potential right ventricular injury from sternal edges, and skin injury due to frequent dressing changes [26]. These concerns contribute to a high threshold for leaving the operating room with an open sternum. To address these limitations, negative pressure wound therapy (NPWT) using VAC systems, which was initially developed for sternal wound infections and mediastinitis, has been increasingly used instead of traditional gauze packing [8,26].

The main rationale for NPWT in an open sternum is to avoid wound contamination through an airtight seal while stabilizing the thoracic cage [26]. However, NPWT must be used cautiously in patients with hemodynamic instability because excessive negative pressure may impair diastolic filling. Eckardt et al. [23] reported outcomes in a mixed population in which persistent mediastinal bleeding was managed using both traditional gauze packing and NPWT. In their protocol, traditional packing was used for cardiogenic shock, whereas NPWT was generally used for coagulopathy. As hemodynamic instability and coagulopathy often coexist, traditional packing may be less effective in controlling coagulopathic bleeding. Lowering negative pressure can provide selective mediastinal compression without causing cardiac tamponade. In a subsequent study from the same institution, NPWT nearly replaced traditional packing and was associated with improved early survival (61% vs. 44% at 6 months) and lower re‑exploration rates (29% vs. 44%) [8].

Before the introduction of commercial VAC systems, Sjögren et al. [1] described a simple negative pressure technique using surgical sponges and chest tubes placed in both pleural cavities to control coagulopathic bleeding. This fundamental concept is similar to that of commercial VAC systems. Our method resembles theirs but uses a separate temporary large-bore chest tube placed directly over the covered gauze to provide more direct suction. The negative pressure generated was sufficient to collapse the packed gauze and enhance selective compression without exerting excessive pressure on the surrounding structures.

Our technique is an adaptation of conventional chest packing and remains rudimentary compared to commercial VAC systems because of the absence of a monitoring console and limited ability to regulate negative pressure. Using a standard three‑chamber drainage bottle, negative pressure typically ranges from 5 to 20 cmH_2_O, although dry suction systems can generate up to 40 cmH_2_O [27]. As shown in Fig. 1, a negative pressure of 20 cmH_2_O was sufficient to collapse the gauze without endangering mediastinal organs. Unlike VAC therapy for infected wounds, which requires prolonged use and precise pressure monitoring, our technique is used for short durations (generally 1–2 days) and gauze collapse can be visually confirmed.

The affordability and accessibility of this technique provide additional advantages. Materials for c‑GSUC cost $33.2, compared with $79.2 for VAC. If multiple dressing changes are required, the cost difference would increase substantially. Using previously published daily maintenance costs [18], the theoretical daily cost of VAC is approximately $111.2, compared with $4.3 for gauze‑based suction. These values represent illustrative cost estimates, as they are derived from fixed daily unit costs rather than from independently observed expenditures.

In our cohort, three patients were diagnosed with mediastinitis. However, two had pre‑existing mediastinitis before being transferred to our institution. Only one patient developed new mediastinitis after delayed chest closure, and the causality with the open sternum was unclear.

Previously reported mortality rates for patients undergoing open sternum and delayed closure range from 20% to 45%, largely reflecting cohorts with hemodynamic instability, mechanical circulatory support, and complex combined procedures [6-9,23,26]. In contrast, only two patients (10.0%) in our study died before discharge. This difference should be interpreted with caution because our cohort consisted primarily of patients with refractory coagulopathic bleeding rather than those with hemodynamic instability. Therefore, the comparison of mortality with historical cohorts is indirect and should be considered hypothesis-generating rather than confirmatory. Most patients in our study underwent chest closure on the day after open sternum application, which is consistent with the short-term indication for mediastinal negative pressure.

This study has limitations owing to its retrospective design and small sample size. The cohort included only patients who underwent this technique for refractory bleeding and not for hemodynamic instability. Because refractory bleeding and hemodynamic instability often coexist, a selection bias may be present. Hemodynamic instability may also confound the assessment of hemostasis in small cohorts. This descriptive study was designed to evaluate feasibility rather than efficacy. Additional multicenter data, including all indications for open sternum, are necessary to evaluate the technique using risk-adjusted or subgroup analyses.

This study describes our experience with a protocolized chest packing technique that generates negative pressure to control refractory mediastinal bleeding. This technique is accessible, inexpensive, and may offer meaningful cost advantages over commercial VAC systems. Although less sophisticated than VAC, it may be a feasible alternative for selected patients.

## Figures and Tables

**Fig. 1.**
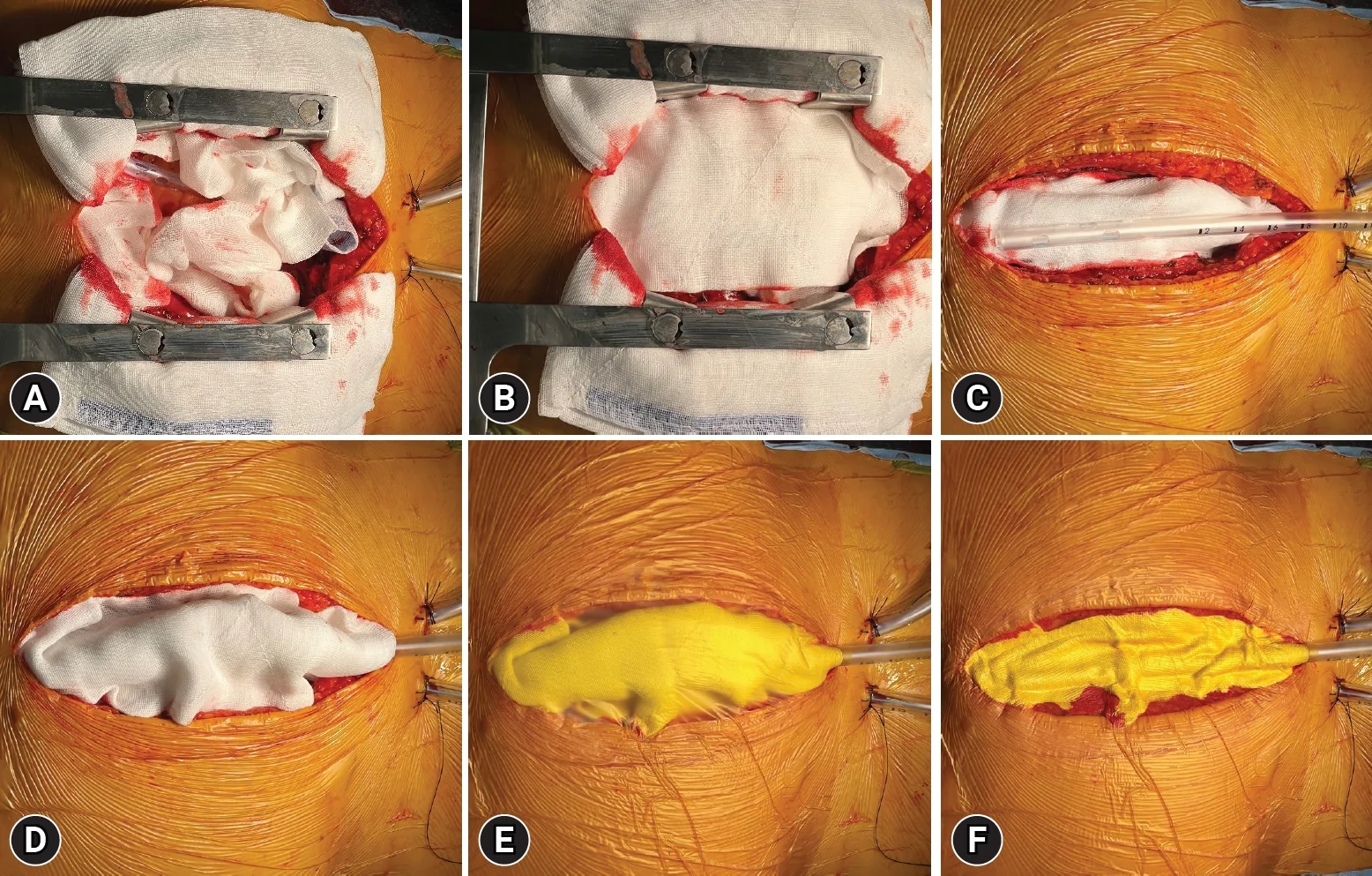
Chest packing with simple gauze and an ordinary chest tube, which generates negative pressure on the mediastinum. The steps used in this technique are as follows. (A) Gauze is sparsely packed around the bleeding area and possible bleeding focus. (B) The entire field is covered with additional gauze to mask the structures. (C) A 32-French regular chest tube is placed on top of the covered gauze. (D) Chest tube side holes are covered using additional gauze. (E) The open wound is sealed with an adhesive drape film (Ioban, 3M Healthcare, St. Paul, MN, USA). (F) The chest tubes are connected to a regular bottle with negative pressure (20 cmH2O) to keep the packed gauze compressed.

**Table 1.** Patient characteristics

No.	Age (yr)	Sex	OPs	Preoperative conditions	Previous OP (n)	Anticoagulation	PLT (×103/μL)	Hb (g/dL)	Hct (%)	aPTT (sec)	PT (INR)	OP time (min)	CPB time (min)	ACC time (min)	Postoperative chest output (mL)	ICU (day)	GW (day)	Complications
1	72	M	AVR, MVR	IE (Elective)	0	Aspirin	138	10.6	29.4	39.0	1.17	540	245	200	740	7	16	None
2	54	M	MVR, Bentall	IE (Elective)	2	None	270	11.2	33.2	39.0	1.19	585	320	300	2,430	10	35	AV block
3	46	M	AVR, AscAoR, CABG	(Elective)	0	Aspirin + clopidogrel	233	11.5	33.9	32.0	1.05	570	330	160	740	17	22	CVA, pericardial effusion, ARF
4	56	F	AVR, TVP, AscAoR	(Elective)	0	None	212	10.3	31.6	39.0	1.05	510	210	90	1,400	8	20	CVA
5	38	M	MVR, TVP, CABG	Behcet (Elective)	2	Warfarin	195	12.8	37.6	51.7	1.41	560	300	258	1,030	5	19	None
6	64	F	AscAoR	(Emergency)	1	None	120	14.2	42.7	36.4	1.22	520	220	137	2,300	6	11	None
7	47	M	Bentall, CABG	Behcet (Elective)	2	Aspirin	170	10.3	29.7	48.3	2.41	825	420	225	940	13	28	None
8	68	M	Bentall, CABG	IE, mediastinitis (Elective)	1	None	117	10.8	32.9	51.9	2.22	720	432	268	3,590	25	25	Pericardial effusion, AKI, wound dehiscence, death
9	76	F	AscAoR, total arch	Emergency	0	None	345	10.4	31.6	35.6	1.32	350	189	111	2,280	6	18	Pleural effusion
10	61	M	Bentall	IE (Emergency)	1	Warfarin	188	8.7	25.3	52.4	2.1	525	282	216	630	3	43	None
11	58	F	AscAoR	(Emergency)	0	None	70	13.2	42.9	31.1	1.02	360	136	76	760	3	12	None
12	53	M	Bentall, AscAoR, Total arch	(Elective)	1	None	126	10.1	30.9	34.6	1.13	460	218	137	1,305	3	13	None
13	56	M	VSRR, AscAoR	Marfan (Emergency)	0	None	234	13.4	40.3	48.0	1.13	420	221	196	2,050	5	17	None
14	50	F	VSRR, AscAoR	(Emergency)	0	None	236	13.9	42.6	33.7	1.02	340	213	164	680	2	9	Pleural effusion
15	70	F	AVR, AscAoR	(Emergency)	0	None	168	11.1	33.5	40.6	1.23	530	258	202	560	6	17	Pericardial effusion
16	44	M	AscAoR, total arch, CABG	(Emergency)	0	None	146	13.6	39.9	39.1	1.23	1,020	481	239	2,760	6	17	AKI
17	86	M	Bentall	IE, (Elective)	1	Aspirin	138	11.6	33.8	40.4	1.12	660	308	199	6,472	8	13	AKI, mediastinitis, wound dehiscence, sepsis
18	65	M	Bentall	Behcet, mediastinitis, (Elective)	2	None	291	9.8	28.9	51.5	1.33	780	344	242	2,062	21	41	Wound dehiscence
19	64	M	AscAoR, total arch	(Emergency)	0	None	105	15.9	47.3	44.2	1.7	470	240	165	1,610	47	53	CVA, AKI, pneumonia, death
20	42	M	MVR, VSRR, AscAoR	Marfan (Elective)	0	None	159	9.1	26.6	30.0	0.97	660	260	234	660	4	15	CVA

M, male; F, female; OP, operation; PLT, platelet count; Hb, hemoglobin; Hct, hematocrit; aPTT, activated partial thromboplastin time; PT, prothrombin time; INR, international normalized ratio; CPB, cardiopulmonary bypass; ACC, aortic cross-clamp; ICU, intensive care unit; GW, general ward; AVR, aortic valve replacement; MVR, mitral valve replacement; IE, infective endocarditis; AV block, atrioventricular block; AscAoR, ascending aorta replacement; CABG, coronary artery bypass grafting; TVP, tricuspid valvuloplasty; CVA, cerebrovascular accident; ARF, acute renal failure; AKI, acute kidney injury; VSRR, valve-sparing root replacement.

**Table 2.** Patient demographics and preoperative details

Characteristic	Data
Demographics	
No. of patients	20
Age (yr)	57.0 (47.8–67.3)
Male sex	14 (70.0)
Anticoagulation	
Aspirin	4 (20.0)
Clopidogrel	1 (5.0)
Warfarin	2 (10.0)
Clinical condition at the time of surgery	
No. of previous surgeries via sternotomy	
0	11 (55.0)
1	5 (25.0)
2	4 (20.0)
Infective endocarditis	5 (25.0)
Connective tissue disease	5 (25.0)
Preoperative laboratory findings	
Platelet counts (×10^3^/µL)	169.0 (129.0–233.8)
Hemoglobin (g/dL)	11.5 (10.0–13.8)
Hematocrit (%)	33.5 (30.3–40.0)
WBC count (/μL)	8,515.0 (6,722.5–13,297.5)
aPTT (sec)	39.0 (35.3–48.0)
PT (INR)	1.0 (1.0–1.0)

Values are presented as number, median (interquartile range), or number (%). WBC, white blood cell; aPTT, activated partial thromboplastin time; PT, prothrombin time.

**Table 3.** Table caption

Variable	Data
Indexed procedure, CABG	4 (20.0)
Valve operation	
Aortic	14 (70.0)
Mitral	4 (20.0)
Tricuspid	2 (10.0)
Aortic surgery	19 (95.0)
Perfusion details, min	
Operation time	535.0 (462.5–660.0)
CPB time	259.0 (218.5–327.5)
ACC time	199.5 (142.8–237.8)
Duration of open chest management, day	1.5 (1.0–2.0)
1	10 (50.0)
2	8 (40.0)
3	2 (10.0)
Chest closure location	
Operating room	18 (90.0)
Intensive care unit	2 (10.0)

Values are presented as number (%) or median (interquartile range). CABG, coronary artery bypass grafting; CPB, cardiopulmonary bypass; ACC, aortic cross-clamp.

**Table 4.** Bleeding characteristics and transfusion details

Variable	Data
Bleeding sites^a)^	
General ooze from an unlocalized area	14 (70.0)
Needle holes in the suture line	8 (40.0)
Posterior to the anastomotic site	3 (15.0)
Intraoperative transfusion (mL)	
pRBC	1,120.0 (800.0–1,600.0)
Platelet concentrate	640.0 (430.0–800.0)
FFP	720.0 (360.0–1,215.0)
Cryoprecipitate	320.0 (140.0–400.0)
Postoperative chest tube output (mL)	1,352.5 (740.0–2,295.0)
Postoperative transfusion (mL)	
pRBC	1,105.0 (445.0–1,537.5)
Platelet concentrate	220.0 (0.0–442.5)
FFP	550.0 (79.8–1,170.0)
Cryoprecipitate	0.0 (0.0–180.0)

Values are presented as median (interquartile range) or number (%). pRBC, packed red blood cells; FFP, fresh frozen plasma.

a) Bleeding focus can overlap in incidence.

**Table 5.** Estimated theoretical costs for c-GSUC vs. VAC

Cost (US$)	c-GSUC	VAC
Initial cost	33.2	79.2
Daily maintenance cost	4.3	111.2
Total theoretical cost	6.3 (4.3–8.5)	166.8 (111.2–222.4)

Values are presented as cost or median (interquartile range). c-GSUC, chest-gauze suction; VAC, vacuum-assisted closure; IQR, interquartile range.

**Table 6.** Postoperative outcomes

Variable	Data
Duration of stay (day)	
Intensive care unit	6.0 (4.3–12.3)
Total hospital stay	17.5 (13.5–27.3)
Death	2 (10.0)
Other complications	
Cerebrovascular accident	5 (25.0)
Pericardial effusion	3 (15.0)
Pleural effusion	2 (10.0)
Acute kidney injury	5 (25.0)
Atrioventricular block	1 (5.0)
Mediastinitis	3 (15.0)^a)^
Wound dehiscence	3 (15.0)^a)^
Pneumonia	2 (10.0)
Sepsis	2 (10.0)

Values are presented as median (interquartile range) or number (%).

a) Two patients with mediastinitis and wound dehiscence at the time of surgery were transferred to our institution.
